# Law and Lunacy: Notes of Recent Cases

**Published:** 1859-10-01

**Authors:** 


### Art. VI.—LAW AND LUNACY: NOTES OF RECENT CASES.

During the quarter which has just expired, three cases came before the Courts arising out of matters relating to Lunacy, all of which, from the amount of public attention drawn to them, deserve to be noted down in the list of "Causes célèbres" in this department of legal inquiry. Each case has one or more points of considerable interest and importance. In the case of *Fletcher* v. *Fletcher*, a plea of admirable ingenuity elicited a very important legal decision, and the subsequent trial at *Nisi Prius* illustrated the sometimes nice distinction between positive delusion and mere obstinate wrongheadedness. The case of *Ruck* v. *Stilwell* (which we give at length in a subsequent portion of the Journal), after occupying the Court for three days, resolved itself into a question as to the meaning of a most ambiguous phrase in an Act of Parliament, the result (at present) being that it has received a construction at the hands of a jury, totally different from that put upon it by men who have taken the Act as their daily guide for fifteen years. The case of Miss Phœbe Ewings shows what an awkward position a medical man may place himself in by humouring a patient; and may also well serve as a caution to medical men as to the mode of conducting the examination of a supposed lunatic.

### Fletcher *v.* Fletcher.

The plaintiff in this action, Edward Philip Fletcher, sued the defendant Edward Charles Fletcher, his uncle, for giving him into custody and causing him to be imprisoned in a lunatic asylum. The defendant pleaded, 1st, the general issue; 2nd, that the plaintiff was a person of unsound mind and incompetent to take care of himself, and a proper person to be taken charge of and detained under care and treatment as a person of unsound mind, and it was unfit, unsafe, improper, and dangerous that he should be at large, and so justified his being placed in an asylum by the defendant; 3rd, that the plaintiff had conducted himself as a person of unsound mind and incapable of taking care of him self, and as a proper person to be detained under due care and treatment, and that a medical certificate of two practitioners had been obtained as required by the 8 & 9 Vict., c. 100, s. 45, and that the defendant had reasonable and probable ground for believing that such certificate was true, and that the plaintiff was of unsound mind. The last plea was demurred to; that is to say, the plaintiff replied that even assuming it to be true, it was no answer to the action, thus raising a question of law for the decision of the Judges. The question having been argued before the full Court in January last, Lord Campbell gave judgment in the following words:—

"I think the plea is clearly bad. At common law only persons who are actually of unsound mind and whom it would be dangerous to leave at large can be restrained of their liberty. Mr. Bovill has gravely argued that persons who *sham* madness may be shut up in lunatic asylums. It would be most dangerous to the liberty of the subject if that were so. There are many *eccentric* persons, as we know from cases of contested wills, who are not by any means to be treated as lunatics. The 8 and 9 Vict. c. 100, s. 99, affords an argument that there is no authority at common law for the proposition that if two men say A. B. is a lunatic, C. D. may take him up and treat him as such."

The practical lesson to be deduced from this decision, is that section 99 of 8 and 9 Vict., c. 100, protects proprietors and officers of asylums acting under proper orders and certificates, but leaves the person *originating* such proceedings to show, as required by the common law, that the person put under restraint is in fact a lunatic.

The questions of fact raised by the first and second pleas came on for trial in July last at the Guildhall, before Lord Chief-Justice Cockburn and a special jury. It was proved that the plaintiff was taken to Kensington House, a lunatic asylum kept by Dr. Wood, on the 16th March, 1858, and there detained until the 30th July, when he made his escape while out walking with a keeper. The following is a copy of the order for his detention given by the defendant.

"I, the undersigned, hereby request you to receive Mr. Edward Philip Fletcher, a person of unsound mind, as a patient into your house. Subjoined is a statement respecting the said E. P. Fletcher.

"Edward Charles Fletcher,
"Lieutenant-Colonel in the Army and Justice of the Peace,
"Kenward Yolding, Kent."

The order was dated the 12th of March, and was directed to Dr. Wood, Kensington House. The statement accompanying the order represented that the plaintiff was twenty-eight years of age, single, of various previous occupations, of the Church of England, late of 4, Brooksby-street, Islington; that the present was not his first attack, but that he had one when he was twenty-six; that he had not previously been under care and treatment; that the duration of the existing attack was uncertain and cause not known; and that he was subject to epilepsy. In answer to the question, "Whether suicidal?" the answer was, "Doubtful; has threatened it;" and to the question, "Whether dangerous to others?" the answer was, "No."

The certificates of insanity were signed by Dr. Gill and Mr. Squire. Dr. Gill stated in his certificate that the plaintiff was a person of unsound mind, &c., upon the following grounds:—

"1. *Facts indicating insanity observed by myself.* General incoherence of manner; peculiar expression of face, the consequence of epileptic fits, the frequency of which satisfied myself of his delusion with regard to money being due to him by the firm to which his late father belonged, and which I ascertained to be delusions by applying to the solicitor of the executor of the estate.

"2. *Other facts (if any) indicating insanity communicated to me by others.* Frequent fits of violence and excitement, accompanied by incoherent expressions, swearing, &c., and his general strangeness of manner, so that the servants absolutely refused to wait upon him; his capricious feelings towards his disreputable associates, begging his landlord one hour to turn them out like a dog, and the next being gentle and familiar with them.

"Dated 14th of March, 1858."

Mr. Squire stated in his certificate that the plaintiff was of unsound mind upon the following grounds:—

"1. *Facts indicating insanity observed by myself.* First: Three several attacks of maniacal excitement, in one of which he made some attempt at self-strangulation, and in two of them was dangerously violent; on one occasion offering to strike me while endeavouring to guard his head from blows upon the table and floor of the room, and being on the other occasion in danger of injuring himself unconsciously against the iron bedstead. Secondly: Delusion that he was engaged to be married to a lady whose property would allow him to prosecute his claims to money that he fancied himself entitled to. On both of these points I satisfied myself of the delusion by seeing the lady and inquiring about the money claims.

"2. *Other facts (if any) indicating insanity communicated to me by others.* An attempt at strangling himself by grasping his throat, and great violence and maniacal excitement observed by Mr. Thomas Jones of the Middlesex Hospital. Secondly: An illusion of blood on the wall, and incoherent expressions.

"Dated 14th March, 1858."

As the action was settled without going into the defendant's case, neither Dr. Gill nor Mr. Squire were examined in support of their certificates.

Plaintiff stated in his evidence that since 1855 he had been subject to severe convulsive fits, which, he said, were like hysteria, and lasted from four to six hours; that in February, 1857, and subsequent to that date, he had made applications to his late father's partners to settle accounts with him. There seems to be no doubt that his father really died largely indebted to the firm of which he was a member, and plaintiff admitted he had been so told by his father's executors, and by a person employed by them to investigate the accounts; but he said that he was not satisfied, and it appeared that his father by his will, made a few months before he died, had purported to leave 5000*l.* to his executors, in trust for the plaintiff, on his attaining twenty-four years of age, besides two other legacies of the like amount to other members of the family. Being examined in reference to Dr. Gill's certificate, the plaintiff said:—

"I had an attack about a fortnight before the 14th of March. It lasted about three hours, and I then perfectly recovered. I have a perfect recollection of what occurred between me and Mr. Gill on the 14th. There was no incoherence of manner. All I said about my claim was that I required an explanation about my father's property. The servants . . . . never refused to wait upon me. I had no disreputable associates that I am aware of. Some of my father's servants were kind to me. Mr. and Mrs. Clark kept a public-house. I often got a meal there. They never called upon me. I never requested my landlord to turn anybody out. It is false. When I was unwell, I said I could not be sure."

In reference to Mr. Squire's certificate, he said:—

"It is not true I ever made any attempt at self-destruction that I am aware of. When I was attacked, I had a feeling of suffocation and a weight on my neck. I put my hand to my cravat. I was sometimes conscious of doing that. I had been engaged to be married to a lady, but I broke off the engagement. At the commencement of 1858, Mr. Squire one day found a book in my room with a lady's name in it. He aske me who the lady was, her age, and asked if it was the party I was engaged to. I said 'No.' He continued chaffing me on the subject. He asked me if I got money, would I make use of it in prosecuting my claim. I said 'Yes.' That was all that occurred that I recollect. I never attempted more than I have stated. On recovering from an attack, for a second or two, everything appeared blood-red. It did not last more than a second or two. I am not aware of any such illusion except when recovering from an attack."

On cross-examination, the plaintiff admitted that in consequence of the interference of a friend, the Commissioners visited him after he had been in the asylum a fortnight; that they examined him, and said his memory was good; that they had seen the accounts, and recommended the Court of Chancery. They refused to discharge him because of his attacks. About the 24th June, the same Commissioners came again to visit the house. He asked them for an interview before they left. They did not seem very willing to grant it, and asked of one of the attendants how he had been. The attendant said, "Not quite so well." After that he was sent for, and stated his case. They did not attend to him a bit.

Mr. Charles Reade, the author of *Never Too Late to Mend,* gave evidence that he had examined the plaintiff after his escape, and found no delusion in his mind.

Mr Morgan said:—

"I am a medical practitioner in Sussex-place, Hyde-park-gardens. I have known the plaintiff twelve years. I visited him in Kensington House in the early part of last July. I came to the conclusion that he was not a fit subject for restraint in a lunatic asylum. I thought it necessary for the plaintiff to have medical supervision. I thought it might be much better carried on under a medical man in the country than in the confinement of a lunatic asylum."

Several non-professional witnesses gave evidence as to their belief in the plaintiff's sanity, and two medical gentlemen who had attended him during some of his attacks, viz., Mr. Bibby and Mr. Langmore, both agreed that they were not strictly epileptic fits. Dr. S. Dickson and Dr. Ruttledge both gave it as their opinion that plaintiff was perfectly sane in August, 1858.

On the conclusion of the plaintiff's case, a conference took place between the counsel, which resulted in an agreement of compromise.

The Lord Chief Justice observed that the defendant had acted on the representations of medical men, and though that would not justify his putting the plaintiff into a lunatic asylum if he was not insane, it took away the imputation of acting from sinister motives. Plaintiff's counsel wished the jury to express their opinion, whether plaintiff was of sound mind.

The jury without hesitation said that he was, in which opinion the Lord Chief Justice concurred.

### Ruck *v.* Stilwell and Another.

The first count in this action was for assaulting and imprisoning the plaintiff in a house called Moorcroft-house, in the parish of Hillingdon, and detaining him there among lunatics and persons of unsound mind, for a period of ten months. The second count alleged that the defendants were keepers of a house licensed for the reception of lunatics, and received the plaintiff upon an order signed by one Mary Ann Ruck, as a private patient, and with two medical certificates; that while the defendants had charge of the plaintiff he recovered, which the defendants well knew, and it was thereupon their duty to transmit notice of such recovery to the said Mary Ann Ruck, but that they neglected to do so, and they also wilfully neglected to transmit notice of his recovery to the Commissioners of Lunacy, but under colour and pretence of the said order and certificates, kept the plaintiff in custody for ten months, &c. To these two counts the defendants pleaded several pleas denying all the material allegations.

The action was tried on the 21st June last, and two following days, at the Guildhall, before Mr. Justice Hill and a special jury

In the course of the trial the plaintiff abandoned the second count, and the case went to the jury on the first count only.

It appeared that the plaintiff was received into the defendant's house on the 5th November, 1857, upon the certificates of Dr. Conolly and Mr. Richard Barnett, M.R.C.S. He was then suffering from delirium tremens, and had delusions respecting the fidelity of his wife. Plaintiff admitted that he continued to hold these delusions until the 25th July, 1858, when they were dissipated by a report made to him by his attorney, Mr. Wainwright, immediately on receiving which he admitted there was no doubt he had been wrong. He was discharged on the 27th August, 1858, on the execution of a Commission of Lunacy, when he was declared to be of sound mind. The history of the cure as given in the evidence is well worthy of perusal, and it will be found in the full report of the trial which we give in a subsequent page; but as it has no bearing upon the point on which the case turned, we need not say more upon the subject here. On the extraordinary character of the medical evidence adduced in behalf of the plaintiff, we commented in our last number.*

The ground on which the plaintiff ultimately rested his case on the first count was, that the medical certificates were informal and insufficient, because—1stly, Dr. Conolly was alleged to be "partly the proprietor of, or a regular professional attendant in, Moorcroft House;" 2ndly, that Mr. Barnett was not a surgeon in actual practice; and 3rdly, that the medical men did not examine the plaintiff apart from each other. In summing up the evidence to the jury, his Lordship (Mr. Justice Hill) made the following observations:—

"A great change had been made in modern times in the mode in which lunatics were treated. Harshness and bodily restraint had given way to gentleness and soothing kindness, and the absence of bodily violence; and one gentleman, Dr. Conolly, had been proved to have taken a prominent part in bringing about this amelioration. But the question now was whether the plaintiff's detention was justified by law. The defendant was the licensed keeper of a licensed asylum, and he said the patient was brought to him with a written order for his reception, and accompanied by two certificates signed by two medical men in the form pointed out by the statute, and that he was justified and bound to take charge of him till he died or was discharged in due course of law. The plaintiff said that there was a provision in the statute which forbade a certificate to be signed by certain parties; that Dr. Conolly's certificate was in violation of the Act of Parliament, and that the certificate was illegal and no justification. It would be a question for the Court whether the certificate, though sufficient in point of form, was sufficient in point of law. The provision was, 'that no physician, surgeon, or apothecary, who or whose father, brother, son, partner, or assistant, is wholly or partly the proprietor of, or a regular professional attendant in, a licensed house or hospital, shall sign a certificate for the reception of a patient into such house or hospital,' &c. (16 and 17 Vict., c. 96, s. 12). The question which he (Mr. Justice Hill) should leave to the jury would be whether they were of opinion

* By typographical errors in the remarks upon this case in our last number, Mr. Canton's name was printed *Carter*, and Mr. Gay's *Gray*.

upon the evidence that Dr. Conolly was 'partly the proprietor of, or a regular professional attendant in, Moorcroft House;' and if so, he should direct them to find a verdict for the plaintiff for such damages as they might be advised. If on the other hand they should be of opinion that he was neither, he (Mr. Justice Hill) should direct them to find a verdict for the defendant. There would also be two other questions, whether Dr. Conolly examined the plaintiff separately and apart from Mr. Barnett, and also whether Mr. Barnett was in actual practice as a surgeon. His Lordship proceeded to observe that there could be no doubt the plaintiff had been suffering from *delirium tremens*, and he (the plaintiff) said that Dr. Conolly and Mr. Barnett were both present all the time he was being examined, but there was no other evidence on that point, and the question would be whether the jury could rely on the plaintiff's evidence in contradiction of the certificates, which stated that the parties had examined him separately from any other medical practitioner. The next question would be whether Dr. Stilwell kept the plaintiff *bonâ fide*, or whether he kept him there for his own gain, and those questions would be very important in considering the question of damages. His Lordship then read the plaintiff's evidence as to his treatment by the defendant, which showed that his delusions continued up to the 26th of July, 1858, and also the evidence of the keepers and Mr. Wainwright on the same point. His Lordship then referred to the book kept at the defendant's establishment, from which it appeared that in the first quarter of the year 1857, Dr. Conolly had received £152 10*s*. from the defendant. His Lordship thought that if Dr. Conolly had been partly the proprietor, he would have received something out of every patient; but the book showed that he only received payments in respect of a certain number. It looked more like the case of a man who was a regular professional attendant in the asylum. In the second quarter ending June, 1857, Dr. Conolly received his consulting fee (25 guineas) and payments in respect of 18 out of 40 patients. In the quarter ending at Michaelmas, 1857, Dr. Conolly received his consulting fee (25 guineas) and payments in respect of 18 patients, in varying sums amounting in all to £184 7*s*. 6*d*. In the quarter ending at Christmas, 1857, he received payments also in respect of 18 patients. So also in the quarter ending in March, 1858, he received his consultation fee (25 guineas) and payments in respect of 18 patients, and in that quarter Mr. Ruck's name appeared with £15 opposite it. In the quarter ending at Midsummer, 1858, Dr. Conolly received his consultation fee of 25 guineas and payments in respect of 18 patients, Mr. Ruck's name having £15 opposite to it. It would be for the jury to say upon that evidence whether Dr. Conolly was or was not a regular medical practitioner in the asylum. His Lordship told the jury that whether they thought Dr. Conolly was in part a proprietor or a regular professional attendant in the asylum, in either case they ought to find their verdict for the plaintiff, with such damages as they might think reasonable, but if they thought otherwise, they ought to find for the defendant."

The jury found (1) that if receiving the money, as shown in the book, made Dr. Conolly a part proprietor—they found the fact of receiving the money; (2) that Dr. Conolly was a regular professional attendant at Moorcroft House, and they assessed 500*l.* damages.

As to Mr. Barnett's not being in practice, the jury found they had not sufficient evidence that he was not, nor had they sufficient evidence to satisfy them that the plaintiff had not been examined separately by Mr. Barnett and Dr. Conolly.

The summing up of the learned judge involves matter of great importance to the medical profession generally; but as the case is still *sub judice,* we refrain from making any comment upon it at present.

### Case of Miss Phœbe Ewings.

The circumstances of this case came before the public upon the execution of a Commission to inquire into the state of mind of Miss Phœbe Ewings. The inquiry commenced at the Castle of Exeter on Friday the 12th of August, and was continued for five days, the Court generally sitting thirteen hours each day. Samuel Warren, Esq., was the Presiding Commissioner.

Miss Ewings, the subject of the inquiry, is nearly eighty years of age. She had an attack of paralysis in October, 1858, which was followed by mania, and it was generally admitted that she had been properly placed in the Haydock Lunatic Asylum in the month of December. There she remained until the 15th of February in the present year, when she was placed in lodgings in Exeter by a distant relative, and under the medical care of Dr. Shapter of that city. In the month of May, a petition in lunacy was presented by a relative, alleging Miss Ewings to be of unsound mind, and praying the appointment of a guardian of her estate. Thereupon Dr. Bucknill was directed by the Lords Justices to visit the lady, and to report to them upon the state of her mind. After examining her at eight interviews, he arrived at the opinion that she was insane. Pending these proceedings, Miss Ewings made two wills, one on the 30th of May, which she afterwards destroyed, and the other on the 2nd of July. In both of these wills Dr. Shapter was named as residuary legatee, and the latter will provided that in the event of his dying in Miss Ewing's lifetime, the property, amounting to nearly £13,000, should go to his eldest son, and in case of his death to other members of Dr. Shapter's family. The solicitor who prepared this will was introduced by Dr. Shapter, and he was present when it was prepared and signed; but within a few days after he wrote to Miss Ewings' solicitor, stating it to be his intention not to take any benefit under the will. He was examined in support of the sanity of the lady, and as might be expected was subjected to severe cross-examination and remarks upon the course he had adopted. It was with great reason contended that his evidence was inconsistent with his treatment.

A chief feature of interest in the case to the readers of this Journal, was contained in the evidence of Drs. Bucknill and Tuke on the part of the petitioner. Although the conclusion at which they had arrived, was also that adopted by the jury, it must be admitted they were very fairly bantered in cross-examination respecting the nature of some of the questions put to the old lady, with the view of testing her mental powers.

Dr. Bucknill stated in his evidence-in-chief, that although Miss Ewings was able to calculate that if she paid 100*l.* a-year for her board, that would be equivalent to 50*l.* for a half-year, and 25*l.* for a quarter of a year, yet she could not tell how much it would be per week. On cross-examination by Mr. Collier, he said that, when he asked her how much she would have to pay per week for her lodgings at 100*l.* per year, she could not answer the question.

*Mr. Collier.*—Will you be kind enough, Dr. Bucknill, to say how much that is per week?

*Witness* (hesitating).—I cannot tell in a moment (laughter).

*Mr. Collier.*—Come, 100*l.* a-year. How much is that per week? Don't be nervous: take time (laughter).

*Witness.*—I decline to tell you.

*Mr. Collier.*—Can you tell without taking out your pencil and going through the figures?

*Witness.*—No (laughter).

*The Commissioner.*—I don't think the learned counsel can (laughter).

*Mr. Collier* (reading from his brief).—It's just 38 shillings and five-twelfths of a penny (laughter). Would not a question of that sort puzzle her?

*Witness.*—It was not done to puzzle her.

*Mr. Collier.*—Did it have the effect of puzzling her?

*Witness.*—No doubt it did.

Dr. Tuke, in his evidence-in-chief, stated, that the general answers of Miss Ewings evinced a want of power to comprehend a subject. He asked, "Who is the present reigning sovereign?" She did not seem to understand it. She did not answer. He said, "Don't hurry yourself; who is the head of the constitution? who administers the law?" He put it many ways, but she seemed not able to understand. The young lady (present) interposed the question, "Who is the present Queen?" Miss Ewings said immediately, "Queen Victoria."

*The Commissioner.*—She beat you in the question, Doctor; yours was rather philosophical (laughter).

On cross-examination by Mr. Collier, Dr. Tuke said:—

I asked her who was the head of the Constitution, who administered the law? I wanted to give her a question that should make her think, but not to puzzle her.

*The Commissioner.*—If you were to ask Mr. Roebuck who was head of the Constitution, he would say the House of Commons (a laugh).

*Mr. Collier.*—So it was to make her think that you asked her who administered the laws. Now, Dr. Tuke, who does administer the laws? (laughter.)

*Dr. Tuke.*—I decline to answer.

*Mr. Collier.*—Why, it is your own question.

*Dr. Tuke.*—Yes. I put it to the lady gently and kindly (laughter).

*Mr. Collier.*—Oh! very well. I will put it to you gently and kindly: Dr. Tuke, who administers the laws of this country? (laughter.)

*Dr. Tuke.*—If I were to answer at once——

*Mr. Collier.*—Oh! take time: consider of it.

*Dr. Tuke.*—I should say the King or Queen. I see the dilemma; but when I put the question to Miss Ewings, I was "talking down" to her.

*Mr. Collier.*—Now, on consideration, should you say the Queen or the Lord Chief Justice of England?

*Dr. Tuke.*—On consideration, I think it might be the Lord Chief Justice.

*The Commissioner.*—Who is the Chief Magistrate? Is it not the Sovereign?

*Dr. Tuke.*—That is my impression. I wanted to ascertain if the lady understood a proposition. If I had asked who is the King or Queen reigning, I have no doubt she would have answered rightly. But my question may have puzzled her, although I put it in every possible way not to puzzle her. I think it possible that the failure in getting an answer may have been the fault of my question.

There can be little doubt as to the worthlessness of such a mode of examining a supposed lunatic for the object in question, and it is much to be regretted in the interest of medical testimony that this evidence should ever have been given in support of the conclusion sought to be established by it. Remembering the opinion current with the public, and nowhere less than in our Courts of Justice, as to the supposed readiness on the part of medical Psychologists to prove people insane, it is of the utmost importance to the credit of the profession that the reasons offered in support of such a conclusion should be such as will bear the strictest scrutiny.

Certain portions of the medical evidence given in support of the sanity of the lady are too curious to be passed unnoticed.

The Commissioner, addressing himself to Mr. Sharp, the ordinary medical attendant of Miss Ewings, previous to her residence at Exeter, and then under examination, said:—

Now the question is, and I have written it down for you, supposing Miss Ewings had yesterday taken the life of another person, and you are this morning asked—if she were being tried for her life—whether she was a rational being and accountable for her actions, would you answer that she was of sound mind, or that she was not?

*Witness.*—I should say she was of sound mind when I saw her, but that having had an attack of mania it might suddenly return, and then of course she would be of unsound mind, but if she had done it while talking to me, then I will say "while in a sound state of mind," but no medical man can say how suddenly an attack of mania may return after it has once appeared. If my memory serves me right, I might say that on the evening of the day when she ran out of the house in an excited state, she was calm in the morning.

*The Commissioner.*—But supposing no attack of acute mania had supervened—of which we have spoken—do you think that if she had killed any one that it would have been "murder?" Would you now sitting in that chair tell the jury—would you say that you believed that she was of sound mind?

*Witness.*—Of sound mind.

*The Commissioner.*—Suppose, with your present knowledge—which you have given to the learned counsel and the jury—you were now to be suddenly informed by some one that she had?

*Witness.*—The answer would be that a sudden attack of mania might have set in, but my impression is that she would not have done it while talking with me, but she might have done it in consequence of a sudden attack of mania having come on.

*The Commissioner.*—Are elderly people very liable to such attacks of acute mania?

*Witness.*—I can't say peculiarly liable to sudden attacks, but I know they may have it.

*The Commissioner.*—Dr. Pritchard, you know, is an authority of some eminence. Listen to this which I read from his book:—"The disease of mental insanity often appears in a more marked and sudden manner in elderly persons who have sustained a slight attack of apoplexy or paralysis, which has perhaps been speedily recovered, and which might be expected to have left traces of the disease. The expectation is verified so far as the sensitive and motive powers are concerned, but the seat of the intellect is found to have been shaken to its very centre." Do you agree with that?

*Witness.*—I don't think that I have sufficient experience to say whether I agree with or differ from that.

Dr. John Andrew Paterson, a fellow of the College of Physicians at Edinburgh, one of the witnesses, in his examination-in-chief, said:—

I am of opinion that Miss Ewings is quite competent to manage the ordinary affairs of life. I should say, subject to the enfeebling of old age, that she was of sound mind. Her memory and perceptive powers were good, and there is nothing about her to indicate lunacy of any kind.

On cross-examination by Mr. Karslake he stated as follows:—

Supposing that she was under a misapprehension as to a man being in the house at Warrington, that may be an exaggerated impression rather than a delusion. My opinion is, that at the time Miss Ewings was removed to the asylum she suffered from acute delirium, and not from mania—the former I believe to be a disturbance of the mind consequent upon physical disease, and the latter a disease of the brain. Often after an attack of mania delusions were discovered. I know that she had an attack of mania; there was nothing unusual in her having the delusions.

*Mr. Karslake.*—Will you give us your definition of delirium and mania? *A.* Yes. *Q.* Do you say that mania is a disease of the brain, or would you not say that the effect on the mind is the result of disease of the brain? *A.* Yes; but we are obliged to draw a line between bodily and mental disease; of course, it is only in that sense we are obliged to draw a line. *Q.* Now, supposing you found a person six months ago had believed, without any foundation, that a person had clasped her around the neck, and had attempted to strangle her, and following upon that she has mania, and is conveyed to an asylum, and six months afterwards she still believes that people attempted to strangle her, for which there is no foundation in fact, would you call that a delusion? *A.* I am not sure I should. *Q.* Would you call it an exaggeration, assuming my premises? *A.* I will assume your premises, but it appears to me that a person would have so little recollection as to make her unable to distinguish between what was a false impression and an actual fact. *Q.* When do you suppose that the impression was caused? *A.* In her mania. *Q.* How are we to draw a distinction of what you call a delusion, or what you call an exaggeration? *A.* I should call it a delusion, but still it is not an existing delusion. *Q.* Then is it a delusion? *A.* It is an unfounded belief. *Q.* What is the difference between a delusion and an unfounded belief? *A.* (hesitating): It is very difficult to say. *Q.* There certainly must be words in the coinage of the English language to express it. What is the difference between an unfounded belief and an unfounded impression? *A.* Because many sensible people may labour under a delusion. *Q.* Supposing that a man was to tell you to-morrow that I am Oliver Cromwell, and nothing on the earth would prevent my belief in it, is that the effect of a delusion or not? *A.* Certainly. *Q.* Supposing that you had been with me an hour, and I told you that a man had come into the room, and had attempted to strangle me, and that nothing of the sort had occurred, is that a delusion? *A.* Yes; I suppose it is. *Q.* It would not be an exaggerated belief? *A.* No; if there had been no foundation for it. *Q.* You would admit that it was, strictly speaking, a delusion? *A.* Yes. *Q.* Are you aware that by the law of the land a person believing in delusions is a madman? *A.* Of course it is entirely a matter of meaning of words. *Q.* No, no, it is not. Are you aware that a person having a persistent delusion, of a thing that does not exist, is insane and incapable of making a will? *A.* Yes; that is an insane delusion. *Q.* I am talking of a delusion as the persistent belief in a thing which does not exist, nor never did exist, would you call that an insane delusion? *A.* It is an insane delusion. *Q.* Then putting the adjective insane, is that the mere putting a vituperative expression to a delusion? *A.* I think almost all people may persuade themselves in the existence of something that does not exist, nor never did exist, and not be mad. *Q.* I grant you that, but is it not an insane delusion? *A.* Of course. *Q.* You recognise a distinction between a mistaken fact and a delusion? *A.* Yes; because a person may believe he is attacked by three persons when it is only one. *Q.* But supposing you had ascertained from Miss Ewings, or she had told you, that at Warrington, before she went to the asylum, a woman with one arm had thrown her down in her lap, and had clasped her round her neck, and you knew that it was not a fact, would not that be a delusion? *A.* I say that I do not believe that a mistaken belief as to what occurred in paroxysms of mania is a delusion. *Q.* Do you believe that delusions engendered during delirium or mania are not delusions? *A.* It is only the memory of a delusion. *Q.* Is it not a continuing delusion? *A.* Certainly not. *The Commissioner:* Supposing that you believed that a black man with three heads bit you, would you believe that to be a delusion? *A.* That is a different thing. *Q.* Supposing a person labouring under insanity had told you that a black man with three heads——*A.* (laughing): I should. *Q.* You will not, Dr. Paterson, attend to my questions. I will try again. Supposing a person labouring under insanity had told you that a black man with three heads bit you very much, and supposing you to have recovered, and that you persisted in the belief that the man had done so, do you mean to tell me that that would be a continuing delusion? *A.* Yes; because it is a very different kind of thing. The difference is just this; that the person is still believing that which is an impossibility, for we know that there is no person with three heads. *Mr. Karslake:* I do not know that.—The Commissioner here told Dr. Paterson that inasmuch as the learned counsel was putting his questions with great care, he trusted he would answer him equally carefully. It was a very important matter.—*Mr. Karslake:* Will you explain the difference between the question the learned Commissioner has put, and the one I have put? *A.* Yes; because it is possible that a woman might have one arm. *Q.* Will you explain the difference between the memory of a delusion, and a delusion? *A.* One is an impression on the mind, and the other arises from a false conception. *Q.* Very well; but supposing that a madman had a delusion that he was told by an angel to murder his father, would you believe that to be a past or existing delusion? *A.* I should say it would be both a past and existing delusion. *Q.* Will you draw the distinction between the two? *A.* It is impossible, because a man would not, in the nature of things, be told such a thing by an angel. *Q.* But suppose that he was told by a gipsy, would that be a delusion? *A.* No; an erroneous belief. *Q.* Define the difference between a delusion and erroneous belief, taking my premises? *A.* It is a belief arising from a false impression of the mind; a totally unfounded impression of the mind. *Q.* Is not that a delusion, as I said before? *A.* I draw a distinction in my own mind as to what in my own judgment is an erroneous belief, or an unfounded belief, or a delusion. I should think that which you mentioned about an angel is the memory of a delusion. *Q.* Give us the definition of a delusion. *A.* It is difficult to say. *Q.* Is the present belief in a non-existent thing a delusion? *A.* Certainly; if you prove to the mind of an individual who holds that belief that the thing never did exist, that is a delusion. *Q.* I assume that one evidence would be that the more you attempt to persuade him that it was a delusion, the more he would persist in believing it? *A.* Certainly. *Q.* Has it occurred to you in your practice to find that a common symptom of mania is the continuing to believe in an exaggerated thing, as distinguished from delusion? *A.* Certainly. *Q.* That is the result of mania? *A.* Very often; for all the mental faculties are exaggerated. *Q.* Now, among all those instances of unfounded belief, have you found that an unfounded aversion to people is a common symptom of insanity? *A.* Yes. *Q.* Have you found that attributing very serious consequences to acts of very minor importance is also a form of insanity? *A.* Yes, and the energies are impaired by paralysis, but I do not say that the cause is existing. *Q.* What is the physical evidence upon which you say that she entirely recovered from paralysis? *A.* Because she has been enabled to walk at a time two or three miles, and could lift dumb-bells. I could see no trace of paralysis. *Q.* Could you see it in her face? *A.* But very vague.

*By the Commissioner.*—Inability to protrude the tongue, as the effect of paralysis, afforded evidence of lesion of the brain.

*Mr. Karslake.*—Will you give me your definition of delirium, and how it differs from mania? *A.* It differs from mania because delirium is the result of bodily disease, whilst mania is the result of disease of the mind. *Q.* Does not mania frequently follow paralysis? *A.* Frequently, I cannot say. *Q.* Do you find that delirium often follows paralysis? *A.* Occasionally. *Q.* Do either of them, as a general rule, follow paralysis? *A.* There is a great suspension of the faculties. *Q.* I believe the forms of insanity are almost innumerable? *A.* Yes. *Q.* And the habits of insane people? *A.* Yes. *Q.* You find that when people are in lunatic asylums they are excessively particular about their religion? *A.* Yes. *Q.* Have you also found that people become excessively particular about religious services? *A.* I think there is occasionally a change of character. *Q.* Have you not found innumerable people who are labouring under delusions? *A.* Yes, I

have. *Q.* And that is an evidence of their insanity? *A.* Yes, in some instances a very strong evidence.

*Re-examined by Mr. Coleridge.*—A person may recover perfectly from an attack of apoplexy or paralysis, and her intellect be as sound as possible.

*The Commissioner.*—Have you ever seen such a case? *A.* I have not.

*Mr. Karslake.*—When you saw Miss Ewings were you told to look for insanity? *A.* Not particularly. *Q.* But when you are told, I suppose you do look for it? *A.* I do.

*Mr. Coleridge.*—Supposing that Miss Ewings stated that a woman with one arm entered her room, and laid her head in her lap, and that afterwards she exaggerated by stating that the woman attempted to strangle her, would you call that a delusion? *A.* I should call it an exaggerated belief.

*The Commissioner.*—Supposing that a person of seventy years of age had lost her sister, and had had an attack of paralysis, and that was followed by an attack of acute mania, so that steps were taken to put her into an asylum, and that you afterwards heard her maintaining certain impressions which had no foundation, would you say that the mind was sound—especially if she still maintained those erroneous impressions up to the period of her eightieth year? *A.*—(abruptly)—I do not think I can give you any other answer than I have given you over and over in this examination. *The Commissioner.*—(warmly)—Really you will not understand me, Dr. Patterson. Do give me an answer. If you found that she still believes to exist, what she believed at the time of the attack of acute mania, does that show unsoundness of mind? *A.* Doubts in the foundation of her belief. *The Commissioner* again repeated the question in several forms, but finding he could not get a proper reply to his question, he said to Dr. Patterson—"Very well, sir, I really cannot make myself understood. You had better go down."

The conclusion of the case was that after a consultation of ten minutes the jury found a unanimous verdict that Phœbe Ewings was not of sound mind.

